# Undergraduate curriculum in palliative medicine at Tampere University increases students’ knowledge

**DOI:** 10.1186/s12904-016-0182-8

**Published:** 2017-01-25

**Authors:** Juho T. Lehto, Kati Hakkarainen, Pirkko-Liisa Kellokumpu-Lehtinen, Tiina Saarto

**Affiliations:** 1 Faculty of Medicine and Life Sciences, University of Tampere, Tampere, Finland; 2 Department of Oncology, Tampere University Hospital, Tampere, Finland; 3 Department of Oncology, Palliative Care Unit, Tampere University Hospital, Teiskontie 35, Rbuilding, 33520 Tampere, Finland; 4 Comprehensive Cancer Center and Faculty of Medicine, Helsinki University Central Hospital, University of Helsinki, Helsinki, Finland

**Keywords:** Undergraduate, Medical education research, Palliative care, Curriculum, Evaluation

## Abstract

**Background:**

Education in palliative medicine (PM) at medical schools reveals wide variation despite the increasing importance of palliative care. Many universities present poor description of the benefits and detailed content of the total curriculum in PM. Using the recommendations of European Association for Palliative Care (EAPC) as a reference, we evaluated the content and outcomes of the curriculum in PM at the University of Tampere, Finland.

**Methods:**

We searched for a PM curriculum by examining the teaching offered by every specialty and compared it to EAPC recommendations. Students’ knowledge was evaluated using a progress test over three consecutive years.

**Results:**

We found 53.5 teaching hours addressing PM issues, which exceeds the recommendation of the EAPC. Basics, symptom management, ethics, and communication skills were well established, while education in psychosocial/spiritual aspects, teamwork and self-reflection failed to reach the recommendations. Out of the maximum of 4.0, the progress test mean scores in PM among the third, fourth, fifth and sixth year students were 0.1 (SD 0.71), 0.69 (SD 1.28), 1.38 (SD 1.46) and 2.53 (SD 1.26), respectively (*p* < 0.001). This growing knowledge was associated with the timely increase in teaching provided through the PM discipline. In addition, the students who completed the optional PM course achieved better mean scores (2.66; SD 1.27) than the others (1.33; SD 1.43) (*p* < 0.001).

**Conclusions:**

The curriculum in PM at the University of Tampere is integrated into the teaching of many disciplines and complied well with the EAPC recommendations. This education led to increasing knowledge in PM among medical students.

## Background

The need for knowledge in palliative care is increasing due to the world’s ageing population and the growing incidence of cancer and other non-communicable diseases [1]. Patients with chronic diseases are becoming increasingly frail and suffer from a wide spectrum of symptoms over months or years before they die. Physicians working in almost all specialties and in many care settings ranging from home and long-term care to outpatient clinics and acute hospitals are often tasked with taking care of these patients.

Despite the increased need for skills in palliative care, medical students and doctors report having insufficient knowledge and training in palliative medicine (PM) [2–4]. Junior doctors often feel unprepared to provide end-of-life care to patients and their families [5–8]. The teaching of PM at medical schools varies widely and is often provided by many disciplines hidden in the syllabus without a clear co-ordination [9–11]. In Europe, no standardized core curriculum in PM exist, and this lack of education is stated as one of the barriers to the development of palliative care [12].

The European Association for Palliative Care (EAPC) regards education in PM for health care professionals to be highly important. Therefore, the EAPC steering group has made recommendations for undergraduate curricula in PM to be utilized at medical schools in Europe [13]. This 40-hour curriculum includes seven main domains of PM integrated in to six sections with a suggested split of the syllabus as well as recommendations for educational strategies and assessment methods [13]. Although many Universities in Europe have a curriculum in PM, their congruence with the recommendations of the EAPC is widely unspecified [9].

In Finland, five universities have a Faculty of Medicine. The University of Tampere has had a chair in PM since 1999 and the University of Helsinki has had one since 2014, while all the other universities lack this professorship. The School of Medicine at the University of Tampere implements a problem-based learning method in a vertically integrated spiral curriculum [14]. The planning of the curriculum was initiated in 1994 when the School of Medicine started to completely innovate its undergraduate medical education. As a new medical subject, PM was gradually integrated into the curriculum following the establishment of PM as a new discipline in 1999. In Finland, Tampere University has thus far been the only university to establish a curriculum in PM.

The aims of this study were 1) to evaluate the undergraduate curriculum in PM at the University of Tampere by using the EAPC recommendations as a reference tool and 2) to evaluate the efficacy of this education by assessing the medical students’ knowledge in PM.

## Methods

### The undergraduate curriculum in PM

Two consecutive professors in PM and two of the authors of this study (JL and TS) evaluated the whole curriculum of the School of Medicine at the University of Tampere. Every teaching blocks and courses offered by different disciplines or specialties were assessed in detail from the electronic learning platform of the medical school. The hours used for each teaching session were calculated and teaching methods were roughly grouped as lectures, interactive seminars, patient contacts, role plays and workshops. The evaluation concerned the year 2013, but no significant changes to the curriculum occurred during the years 2014 and 2015. In addition to the obligatory studies, the optional course of PM established in 2012 was evaluated separately.

The EAPC recommendations for the development of undergraduate curricula in PM at European Medical Schools and the Palliative Education Assessment Tool (PEAT) were used as a reference to assess the content and mapping of the teaching of PM issues at the University of Tampere. The seven domains presented in the PEAT were integrated into six sections (basics of palliative care, pain and symptom management, psychosocial and spiritual aspects, ethical and legal issues, communication, and teamwork and self-reflection) as described by the steering group of the EAPC [13, 15].

### The benefit of teaching

The cumulative learning of the medical students at the University of Tampere is evaluated by a progress test based on a single best answer format [16, 17]. All students from the first to the final year of medical school perform the same test three times a year and they must participate in 80% of the progress tests arranged during their medical school studies. The test consists of 175 multiple-choice-questions based mostly on clinical vignettes with three to five alternatives and only one correct answer (single best answer items). The student gets one point for a correct answer, zero point for leaving the question unanswered and loses 0.5 point for a wrong answer. The entire test is performed within three hours. All fields of medicine are covered, meaning that the blueprint contains 12, 7 or 4 items per subject matter; PM is among those given 4 items/test. These four questions concerning PM (hypothetical patient cases) are prepared by PM professor. Cumulative knowledge in PM was evaluated by the answers to these questions by different year students at the medical school and among the students who had and had not participated in the optional course. We chose the progress tests performed in February in three consecutive years (2014–2016) for the evaluation because February is the last testing point for graduating students of the sixth year and thus represents the achieved end point. The tests arranged in February also best differentiate the amount of teaching in PM among different year students.

A structured evaluation of the students’ opinions on PM education was achieved after a two-day (10 hours) teaching session of symptom management provided by the discipline of PM. Every student underwent this largest teaching session of PM during the spring term of their fifth year or the autumn term of their sixth year depending on the group to which they belonged. This interactive seminar was performed in groups of eight to ten students and included a patient interview in a hospital ward with a treatment plan for palliative care and feedback discussions. Evaluations from students were gathered through a web-based questionnaire after the teaching session.

### Statistical analyses

Results from the progress test scores were described by means and SDs. The Mann–Whitney U-test was used for comparisons of progress test results between different groups of students. A two-sided *p*-value of less than 0.05 was accepted as statistically significant. Analyses were done using SPSS version 23.0 software.

## Results

### Palliative medicine curriculum

The undergraduate curriculum of the School of Medicine at the University of Tampere contained a total of 53.5 hours of teaching in PM issues. The topics, teaching methods, responsible specialties and the durations of each teaching session are presented in Table 1.

**Table 1:** Undergraduate curricula of palliative medicine in the University of Tampere

Year	Title (Section in the EAPC syllabus)	Specialty	Teaching method	Hours
1	Introduction to medical ethics (E)	Medical ethics	Interactive seminar	3
2	Physiology and pharmacology of pain (S)	Pharmacology	Lecture	1
	Communication skills (C)	General medicine	Lecture, Workshop	4.5
	The end-of-life (B)	Geriatrics	Lecture	1.5
3	Basics of palliative care (B)	Palliative medicine	Lecture	0.5
	End-of-life care (B)	Palliative medicine	Interactive seminar, Patient contacts	1.5
	Basics of cancer pain management (B)	Palliative medicine	Lecture	0.75
	Euthanasia (E)^a^	Palliative medicine	Interactive seminar	3
	Communication skills (C)	General medicine	Role play, Workshop	3
4	Facing the family of dying patient (C)	General medicine	Workshop	2
	Ethics in decision making (E)	Anesthesiology	Lecture	0.75
	Fatigue in cancer patient (S)	Palliative medicine	Lecture	0.75
	Chronic pain (S)	Neurology	Lecture	0.75
	Pain and analgesics (S)	Pharmacology	Lecture	1.5
	Pain (S)	Pharmacology	Interactive seminar	1.5
5-6	Symptom management in palliative care (S)	Palliative medicine	Interactive seminar, Patient contacts	10
	Social security in cancer patients (P)	Oncology	Lecture	0.75
	Psychological crisis in cancer (P)	Oncology	Lecture	0.75
	End-of-life care in elderly (S)	Geriatrics	Lecture, Patient contacts	3.5
	Constitution of a human (P)	Geriatrics	Lecture	1.5
	Pain (S)	Anesthesiology	Workshop	1.5
	Neuropathic pain (S)	Neurology	Interactive seminar	1.5
	Palliative medicine (S)	Palliative medicine	Interactive seminar	5
	Alternative medicine (S)	Palliative medicine	Interactive seminar	3

*B*: Basics of Palliative Care; *S*: Pain and symptom management; *P*: Psychosocial and spiritual aspects; *E*: Ethical and legal issues; *C*: Communication. ^a^Teaching session removed from the curriculum in 2014

The discipline of PM was responsible for 24.5 hours (46%) of the total education. The remainder of the content was integrated into the syllabi of seven other specialties. Palliative medicine was integrated early into preclinical studies, but 51% of the total education and 73% of the teaching provided by the PM discipline was conducted during the clinical studies of the last two years at the medical school. Only 10.5 hours (20%) of the teaching was conducted purely through lectures, while all the other sessions included interactive or experiential methods (e.g., patient encounters in hospital wards or hospice).

### Comparison of the curriculum to EAPC recommendations

The comparison between the PM curriculum at the University of Tampere and recommendations made by the EAPC is presented in Table 2. Over half of all the teaching at Tampere University dealt with pain and symptom management, which exceeded the EAPC recommendations. By contrast, psychosocial and spiritual aspects together with teamwork and self-reflection received less educational attention than recommended by the EAPC.

**Table 2:** The split within the syllabus in the undergraduate curriculum of palliative medicine according to the recommendation by the EAPC and in the University of Tampere

Recommendation by EAPC Syllabus^a^	Recommendation by EAPC Hours (% of total)	University of Tampere Hours (% of total)
Basics of palliative care	2 (5)	4.25 (8)
Pain and symptom management	20 (50)	30 (56)
Psychosocial and spiritual aspects	8 (20)	3 (6)
Ethical and legal issues	2 (5)	6.75 (12)^b^
Communication	6 (15)	9.5 (18)
Teamwork and self-reflection	2 (5)	0 (0)
Total	40	53.5

*EAPC*: European Association for Palliative Care; ^a^See ref. number 13 for detailed information; ^b^3.75 h since year 2014

### Optional course in palliative medicine

In addition to the obligatory studies, an optional course in palliative medicine entitled “Key elements of high-quality palliative care in clinical practice” has been offered since 2012 for students from the fourth year of medical school. The course consisted of nine lectures (13.5 hours), 10 workshops (15 hours), two interactive case-seminars (3 – 4 hours), a patient interview with a case study (12 hours) and self-learning for workshops (20 hours). Teaching was planned to provide advanced knowledge in symptom management, communication, and psychosocial support. The syllabus of the optional course from 2014 is presented in the Appendix 1. The syllabus has remained largely the same since 2012.

### Progress test

**Fig. 1:**
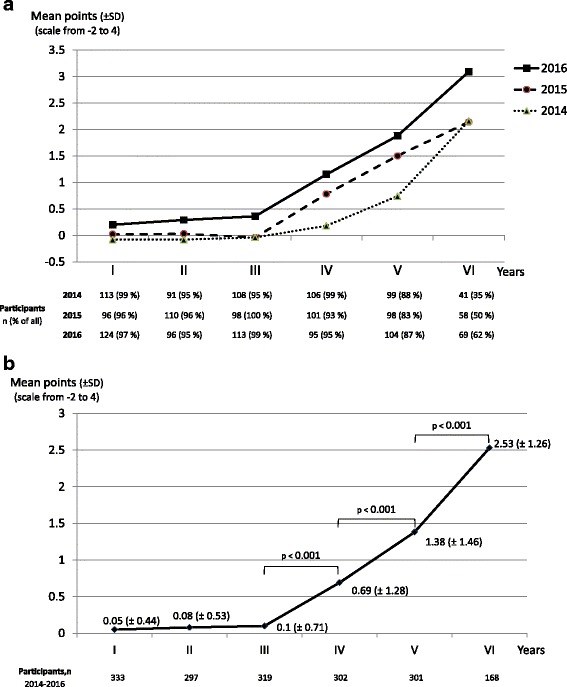
Progress test results from the questions concerning palliative medicine in students from first to sixth year of medical school. **a** Mean scores in the years 2014-2016 (number of participants and their proportion of all the students are shown below the chart). **b** Combined mean scores from the years 2014-2016 (total number of participants are shown below the chart)

Results from the progress test performed in February 2014, 2015 and 2016 are shown in Fig. 1. Three examples of the case scenarios concerning PM are presented in Appendix 2. By February, all graduating students in their sixth year in medical school had received all their PM education (Table 1). Of the fifth-year students completing the progress test, 24 (8%) had participated in the 10-hour seminar “symptom management” and none in the other 5-hour interactive seminar on PM (Table 1).

**Fig. 2:**
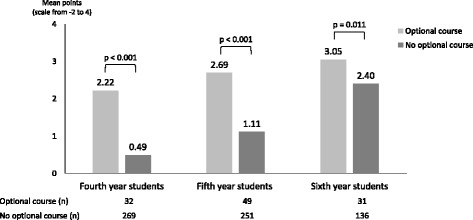
Combined progress test results in three consecutive years (February 2014-2016) from the students who had and had not participated in the optional course of palliative medicine

Students who had undergone the optional course in PM achieved significantly better results in the progress test compared to students who had not (Fig. 2). As a whole, the fourth to sixth year students with and without the optional course received a mean of 2.66 (SD 1.27) and 1.13 (SD 1.43) points, respectively (*p* < 0.001).

### Feed-back from students

**Fig. 3:**
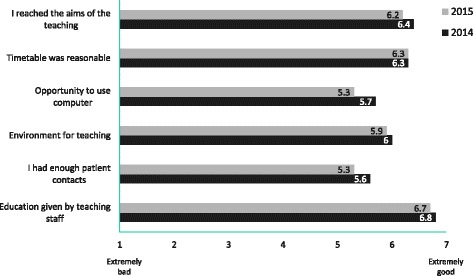
Evaluation of the interactive small group seminars provided by the discipline of palliative medicine (Symptom management in palliative care) during 2014 (*n* = 104) and 2015 (*n* = 104). Mean numbers on a scale from extremely bad (1) to extremely good (7)

Results from the structured feed-back questionnaire on the two-day (10 hours) teaching session (Symptom management in palliative care) is presented in Fig. 3. All aspects received over five points on a scale from 1 – 7 with the highest points achieved in the questions concerning the actual teaching.

## Discussion

We described the curriculum of PM at the University of Tampere, which integrated the education offered in many specialties in addition to the discipline of PM and compared favorably to the recommendations of the EAPC. Medical students demonstrated increasing knowledge in PM during their studies and they found the teaching very valuable.

In Finland, only the University of Tampere has a formal curriculum in PM thus far, while the other four universities with medical faculty are in the planning phase of a PM curriculum. The teaching in palliative care has increased at medical schools in many countries, but its availability still differs between universities [18, 19]. A recent study from 43 European countries demonstrated that 28 (65%) of the countries include PM in the curriculum of at least one of its medical schools, but teaching in PM in all universities was compulsory in only six (14%) of the countries [19].

Many medical schools report teaching PM, but the exact content of this education is either unknown or differs markedly [9, 18, 20]. In the medical schools of the USA, education in PM ranges from 2 hours of lecturing to weeks of patient contacts and clinical training [11]. A recent systematic review by DeCoste-Lopez et al. revealed a similar variation in the length and contents of PM education around the world, including reports from Europe. Notably, many of the curricula were not described in enough detail to achieve even basic information about the palliative care topics covered [20]. Our study shows that the curriculum in PM at the University of Tampere is compliant with the recommendations made by the EAPC. In addition to lecturing, the teaching also included interactive and experiential methods such as patient encounters in hospital wards and hospice, which are also supported by the EAPC [13]. In fact, after the addition of the two-days teaching session (Symptom management in palliative care) in 2012, the total hours of teaching issues of PM exceeded the recommended curriculum. This was especially true for pain and symptom management, while education concerning psychosocial and spiritual aspects together with teamwork and self-reflection did not meet the criteria of the EAPC. Psychosocial aspects are of great importance when facing patients with incurable diseases. Additionally, working in multidisciplinary teams is mandatory in the field of PM. Therefore, these aspects should scrutinized when both developing our formal nationwide core PM curriculum and ensuring the provision of a true holistic undergraduate curriculum without overloading it with symptom control issues.

We found that at the University of Tampere different palliative care issues are taught by many other specialties in addition to the discipline of PM. This is important since symptom-centered care should be integrated in the treatment of every patient suffering from incurable disease (so-called horizontal integration). The amount of teaching concerning issues of PM increased closer to graduation from our medical school. Preclinical studies concentrated in the basics of palliative care and pain management, while broader knowledge in PM was achieved during clinical studies (so called vertical integration). This horizontal and vertical integration is also found in some other universities and is recommended by the EAPC [13, 21]. However, a formal curriculum in PM is needed to co-ordinate teaching between many specialties and to optimize the content of teaching to ensure the overall knowledge and experience of the students.

We included all teaching from the electronic learning platform with topics and content concerning PM. However, there was no detailed cross-linking of single issues between different teaching lessons, and there might still be training hidden in the syllabi, since some teaching, such as discussions of patient cases in problem based learning and in clinical rounds on wards, probably contained aspects of PM. Thus, our evaluation offers a good overview of the curriculum in PM but has limitations in giving a detailed description of all PM issues covered in the teaching.

Earlier studies have revealed increased skills in palliative care among medical students after receiving education in PM [22, 23]. A recent study by Gerlach et al. showed that a course consisting of seven times 90-minute classes in palliative care increased medical students’ self-confidence concerning somatic, spiritual and psychological aspects of PM and resulted in high knowledge in a post-course test [24]. Education in PM may also help to adapt patient-centered care in general [25]. However, most of these studies have concentrated on the short term effects of a single course of PM education [20, 23, 24]. We tested the knowledge among medical students at different times in their studies through the whole curriculum in PM. The increase in the knowledge measured by a multiple-choice progress test started after the first teaching sessions provided by the discipline of PM during the third year and continued through the largest teaching sessions in PM in fifth and sixth years of medical school. Although we suggest that this increase in knowledge was mainly due to the increased teaching in different aspects of PM, it may also have been bolstered by more student encounters with patients suffering from incurable diseases during their studies at medical school. In addition, the students who had taken part in the optional course in PM achieved better results when compared with the others. The difference was largest among the fourth and fifth year students, when the obligatory teaching of PM was still sparse but remained significant among the graduating students.

Although our results clearly demonstrate the benefits of teaching PM, we acknowledge some limitations in our evaluation. First, only about half of the sixth-year students took part in their last progress test in February 2014–2016. Although their knowledge in PM increased and the effect of the optional course remained until sixth year at medical school, the final benefit of the curriculum at time of graduation and later is slightly uncertain. Second, students during their last years at medical school might have achieved some general experience in predicting the right answers on multiple choice tests compared to younger students. However, as the questions were changed in every progress test and as the students lost 0.5 point for a wrong answer, we suggest that trying to guess the right answer was uncommon. Third, progress tests do not evaluate all aspects of palliative care, as they are more suitable for verifying medical knowledge than qualities such as psychosocial caring, ethics and communication skills. Further studies are needed to evaluate the long-term effects of the whole PM curriculum after graduation. Such an evaluation should try to include patient outcomes and behavioural aspects, including communication skills and empathy assessments in addition to symptom control issues, as suggested in other reports [13, 22, 24]. The impact of the curriculum on students’ career choices and participation in undergraduate education in PM should be further studied as well.

Feedback concerning the teaching of PM was achieved from the largest session (Symptom management in palliative care). Although this covers only about one-fifth of all the teaching in PM, the results reflect the positive views of the students. The quality of the teaching and its benefits in terms of achieving its aims were graded especially high. In Finland, medical students are allowed to work as junior doctors after their fourth year at medical school. Thus, most of the students had been challenged by caring for a dying patient before this teaching session, which probably increased their motivation for PM education. The other explanation could be the interactive nature of teaching in small groups instead of simple lecturing. Basics in PM should be taught early, but we suggest that more advanced learning is best achieved when its clinical context is understood. A formal assessment of the learning needs of the students, especially after their first contacts with dying patients, might be helpful for further development of the curriculum. Our results are in line with previous studies showing that education in PM is appreciated by medical students as well as by physicians [25–27].

## Conclusion

The curriculum of PM at the University of Tampere in Finland shows integration between many disciplines. It is in line with the recommendations of the EAPC and includes a broad education in symptom management, but needs development in psychosocial and spiritual aspects. The benefits of the curriculum for the students is demonstrated by their increasing knowledge in PM and positive feedback regarding the teaching. The curriculum evaluated in this study could be used as a basic model when developing a core curriculum of PM in Finland. A similar evaluation in all medical faculties is recommended to explore the effectiveness of the curriculum, to compare students from different medical schools and to enable further development of PM education.

## Appendix 1

The syllabus of the optional course in palliative medicine entitled “Key elements of high-quality palliative care in clinical practice” at the University of Tampere in 2014.

- Introduction to palliative medicine and advanced care planning
  - Lecture (1.5 hours)
- Cancer pain
  - Lecture (1.5 hours) and workshop/case-seminar (1.5 hours)
- Gastrointestinal symptoms
  - Lecture (1.5 hours) and workshop/case-seminar (1.5 hours)
- Respiratory symptoms and palliative care in respiratory diseases
  - Lecture (1.5 hours) and workshop/case-seminar (1.5 hours)
- Palliative care in cancer
  - Lecture (1.5 hours) and workshop/case-seminar (1.5 hours)
- Palliative care in internal medicine and end-of-life care at home
  - Lecture (1.5 hours) and workshop/case-seminar (1.5 hours)
- Communication
  - Workshop (1.5 hours)
- End-of-life care
  - Lecture (1.5 hours) and workshop/case-seminar (1.5 hours)
- Palliative care in neurological patients
  - Lecture (1.5 hours) and workshop/case-seminar (1.5 hours)
- Fatigue and cachexia
  - Workshop/case-seminar (1.5 hours)
- Palliative care in dementia
  - Workshop/case-seminar (1.5 hours)
- Psychosocial aspects in palliative care and treatment of mental symptoms
  - Lecture (1.5 hours)
- Seminars of the students own case presentations with discussion and feedback
  - Students interviews and examines their own teaching patient and prepare a case presentation with advanced care plan
  - Workshop/case seminar (2 × 2 hours)

## Appendix 2

Examples of the four questions concerning palliative medicine out of the 175 questions in progress-test in February 2014, 2015 and 2016 with single best answers.

### Example question from February 2014

Seventy-eight years old man with severe end-stage COPD comes to you with wheel-chair. Despite the maximal COPD-medications and rehabilitation he suffers from severe episodes of breathlessness. He is able to walk only couple of meters indoors. His oxygen saturation is 95% on room air and carbon dioxide levels have been normal in blood.

Which one is the primary way to enhance the symptomatic care of breathlessness?

Choices:

A) I send the patient for initiation of palliative oxygen therapy
B) I start small-dose opioid (e.g. morphine)
C) I start regular corticosteroid p.o.
D) I start small-dose benzodiazepine (e.g. lorazepam)

*Single best answer: B*

### Example question from February 2015

Forty-eight years old man has a pancreatic cancer with metastases in liver and lungs. He has taken paracetamol 1 g thrice a day, but still has severe constant pain (VAS 7).

Which one is the primary way to improve the pain medication?

Choices:

A) I start combination of paracetamol and codeine
B) I start ibuprofen 600 mg thrice a day
C) I start long-acting morphine 30 mg twice a day and short-acting morphine 10 mg as needed for breakthrough pain
D) I start short-acting morphine 10 mg thrice a day

*Single best answer: C*

### Example question from February 2016

A patient with renal cancer and liver metastases has started oxycodone for pain one week ago. The dose is now 60 mg twice a day and the pain is in control with this. This patient comes to you due to constipation.

What is best to do? Choices:

A) to follow the patient as the constipation caused by oxycodone shall pass
B) to lower the dose of oxycodone to relieve the constipation
C) to start osmotic or stimulant laxative
D) to advice the patient to use enemas
E) to switch oxycodone to tramadol

*Single best answer: C*

## Abbreviations

PM: Palliative medicine
EAPC: European association for palliative care
SD: Standard deviation
PEAT: The palliative education assessment tool
SPSS: Statistical package for the social sciences
